# Active Child, Accomplished Youth: Middle Childhood Active Leisure Fuels Academic Success by Emerging Adulthood

**DOI:** 10.3390/children11091140

**Published:** 2024-09-20

**Authors:** Laurie-Anne Kosak, Kianoush Harandian, Simon L. Bacon, Caroline Fitzpatrick, Luca Correale, Linda S. Pagani

**Affiliations:** 1 School of Psycho-Education, University of Montreal, Montreal, QC H3T 1J4, Canada; laurie-anne.kosak@umontreal.ca (L.-A.K.); kianoush.harandian@umontreal.ca (K.H.); 2 School Environment Research Group, University of Montreal, Montreal, QC H3T 1J4, Canada; caroline.fitzpatrick@usherbrooke.ca; 3 Montreal Behavioural Medicine Centre (MBMC), Centre Intégré Universitaire de Santé et de Services Sociaux du Nord-de-l’Île-de-Montréal (CIUSSS-NIM), Montreal, QC H4J 1C5, Canada; simon.bacon@concordia.ca; 4 Department of Health, Kinesiology and Applied Physiology, Concordia University, Montreal, QC H4B 1R6, Canada; 5 Department of Preschool and Elementary School Education, Université de Sherbrooke, Sherbrooke, QC J1K 2R1, Canada; 6 Sports Science Unit, Department of Public Health, Experimental and Forensic Medicine, University of Pavia, 27100 Pavia, PV, Italy; luca.correale@unipv.it; 7 Sainte-Justine’s Hospital Research Center, Montreal, QC H3T 1C5, Canada

**Keywords:** active leisure, physical activity, organized sport, school achievement, child development, longitudinal analyses

## Abstract

**Background/Objectives**: Physical activity is an important protective factor throughout life. However, little research has observed the associations between the practice of physical activity and academic success longitudinally, and none have done so with a pan-Canadian sample. This article aims to examine the prospective associations between active leisure in middle childhood and academic achievement in emerging adulthood, for both boys and girls, beyond several family factors. **Methods**: Participants are 2775 children from the National Longitudinal Study on Children and Youth (NLSCY) aged between 12 and 20 years. Active leisure was self-reported by children at age 12 years regarding their weekly organized sport, artistic sport, and unstructured physical activity participation outside of school hours. Academic success was measured by self-reported school average at age 18 years and the obtention of high school diploma at age 20 years. **Results**: Girls who engaged in more organized or artistic sports at age 12 years had better academic results at age 18 years (respectively β = −0.082, *p* < 0.01; β = −0.228, *p* < 0.001). Both boys and girls who partook in more organized sports at age 12 years were more likely to graduate from high school by age 20 years (respectively β = −0.146, *p* < 0.001; β = −0.071, *p* < 0.05). However, girls who engaged in more unstructured physical activity at age 12 years had lower academic achievement at age 18 years (β = 0.077, *p* < 0.001). **Conclusions**: Policy makers should aim to reduce the many barriers to an active lifestyle in childhood. Parents should be encouraged to lead their children to go play outside with friends to allow them to fully reap the benefits of an active lifestyle from a young age.

## 1. Introduction

An active lifestyle has many short- and long-term benefits for physical health [1]. Active people also have better cognitive functioning and mental health [2,3]. Although the many benefits of physical activity are well established, only a minority of children meet the recommendations for daily movement [4]. These children who do follow movement guidelines have better academic achievement, fewer socio-behavioral problems, and better emotional functioning [5].

While all children exercise in their physical education classes, few take part in physical activities outside of school hours [6]. The ubiquity of screens encourages sedentary time in middle childhood, reducing active play opportunities by the end of primary school [7]. As children are swapping bicycles for phones, they are losing many of the lifelong benefits of an active lifestyle.

For children, active leisure represents any type of physical activity that is done outside of school hours. It encompasses organized sport, like team sports with an instructor, and any type of play involving movement, like cycling or kicking a ball with friends. Different types of physical activity have different benefits [8]. While the benefits of physical activity have been widely studied, surprisingly little research has looked at the relationship between middle childhood active leisure and ulterior academic success.

Physical activity generally has a positive impact on child and adolescent ability to succeed, as shown by greater cognition [9] and performance in mathematics [10]. Children who follow daily movement recommendations perform better at school [11]. In a study examining the influence of teamwork in sports on academic achievement, Fox et al. [12] have found that, for girls, both physical activity and team sports were independently linked to better academic performance, while for boys, only team sports influenced this outcome. A scoping review found a generally positive impact of physical activity on academic performance in adolescents [8]. Donnelly et al. [13] found conflicting results regarding the possible impact of physical activity on academic achievement. While multiple studies have looked at physical activity and academic achievement, there is no consensus on the types or level of physical activity that might impact achievement, and while many studies found a positive effect, others found a negative effect of physical activity on student achievement [13].

Human Capital theory suggests that people who possess more skills can more easily learn new ones, and thus have better chances of future success [14]. Sports and physical activity in general develop a variety of skills that could contribute to future success, such as leadership, attention, and resilience [15,16,17]. Children who play sports with a coach also show more entrepreneurial skills [18]. Thus, physical activity represents an accessible means of developing various types of Human Capital (Emotional, Financial, Individual, Intellectual, Physical, and Social) [19]. As a component of lifestyle, children who partake in more active leisure might develop these skills and apply them to the classroom. This could lead to higher achievement in school, which in turn could lead to lifelong success [19].

There are conflicting studies on the possible impacts of engaging in physical activity on school achievement [12]. Little longitudinal research has been conducted. Physical activity practice during childhood had a positive effect on academic performance in pre-adolescents [9]. These associations were also replicated longitudinally, albeit over a short period. Participation in sports is associated with better language and math scores a year later [10]. Consequently, it is difficult to identify whether physical activity influences academic success, or whether it is the other way around. Moreover, sex is often treated as a control variable, although boys and girls experience both participation in sports and academic achievement in vastly different ways [20,21]. Notably, boys are placed in sports at a younger age by their parents, while girls are more often oriented towards other types of leisure activities [22]. Girls also often do better than boys in school, which is partly due to their earlier cognitive maturation [23].

Using a longitudinal population-based birth cohort design, the objective of this study is to assess the long-term benefits of extracurricular physical activities in childhood on later school achievement. More specifically, the purpose of this study is to examine the prospective associations between organized sports, unstructured physical activity, and artistic sports at age 12 years, and average grades and high school completion at ages 18 and 20 years, for both boys and girls, beyond family characteristics. We hypothesize that partaking in more active leisure in middle childhood will lead to higher grades and a greater chance of having completed high school in emerging adulthood, above and beyond control factors.

## 2. Materials and Methods

### 2.1. Participants

Participants are from the National Longitudinal Survey of Canadian Children and Youth (NLSCY). The NLSCY is a nationwide longitudinal dataset using a sequential multiple cohort design that was developed by Human Resources Development Canada and Statistics Canada (https://www23.statcan.gc.ca/imdb/p2SV.pl?Function=getSurvey&Id=3513 (accessed on 13 September 2024)). Cycle 1 data were collected in 1994–1995 from a randomly selected birth cohort from households surveyed by Statistics Canada’s Labour Force Survey (LFS) and yielded 22 831 participants aged 0 to 11 years. Selected children were followed biennially, and more participants aged 0 to 2 years were added each cycle. Residents living on Indian reserves, Crown lands, institutions, remote regions, and full-time members of the Canadian Armed Forces were excluded from the survey, as they were not included in the LFS. Data were collected via computer-assisted interviews with the person most knowledgeable (PMK) of the child. Paper questionnaires were distributed to the children from age 11 years. In most cases (88.9%), the biological mother served as PMK. Data were collected over a 9-month period for each cycle.

Participants for this study are 2 775 children (50.4% boys) who were 12 years old at Cycle 2, Cycle 3, and Cycle 4 and pooled their data on selected variables. Thus, the data from the first time point of the study were collected between 1996 and 2001.

### 2.2. Measures: Childhood Active Leisure Predictors (Age 12 Years)

Children reported on their active leisure venues on three items representing *organized sports*, *unstructured physical activity*, and *artistic sports*: “In the past year (12 months), how often have you: (a) Played sports with a coach or instructor, other than in gym class? (school teams, swimming lessons, etc.)? (b) Played sports or done physical activities without a coach or an instructor (biking, skateboarding, etc.)? and (c) Taken part in dance, gymnastics, or cheerleading groups or lessons, other than in gym class?”. The possible responses were: 1 = never, 2 = less than once a week, 3 = 1 to 3 times a week, or 4 = 4 or more times a week.

### 2.3. Measures: Indicators of School Achievement (Ages 18 Years and 20 Years)

School achievement was measured using two self-reported variables: average grades (age 18 years) and high school completion (age 20 years).

*Average grades* were reported by asking: “Overall, what is your average mark this year? (give best estimate)” (1 = 90% to 100%, 2 = 80% to 89%, 3 = 70% to 79%, 4 = 60% to 69%, 5 = 55% to 59%, 6 = 50% to 54%, 7 = 50% or less). Categories 50% to 54% and 50% or less were combined because of the small number of participants.

*High school completion* was determined by asking: “Have you received or completed the requirements for a high school diploma or its equivalent?”. Answers were coded as 0 = obtained high school diploma and 1 = did not obtain high school diploma.

### 2.4. Measures: Adjustment for Baseline Academic Achievement and Family Characteristics (Age 12 and 14 Years)

*Baseline academic achievement* was reported by teachers by asking: “Compared to the other students in the class you teach this student, how would you rate this student’s current academic achievement?”. Answers were standardized and coded as 0 = “Near the middle of the class”, “Above the middle of the class but not at the top”, or “In the middle of the class”; and 1 = at risk, “Below the middle of the class but above the bottom”, or “Near the bottom of the class”.

*Family configuration* was derived from basic demographic information. A family is considered intact when the couple is either married or common-law, and where children are the natural and/or adopted offspring of both members of the couple (0 = intact family, 1 = non-intact family).

*Income adequacy* was computed from household income reported by PMKs into five categories: lowest, lower-middle, middle, upper-middle, and highest (0 = middle, upper-middle or highest, 1 = lowest or lower-middle).

*Maternal education* was reported by PMKs (0 = graduated high school, 1 = did not graduate high school).

*Maternal depressive symptoms* were reported by PMKs and assessed with a 12-item abridged version of the original 20-item CES-D depression rating scale (α = 0.82) [24]. Final scores ranged from 0 to 36, where a higher score indicates the presence of more depressive symptoms. Scores were standardized and computed as 0 = below the mean or 1 = above the mean.

*Family dysfunction* was assessed using the 12-item McMaster Family Functioning scale (α = 0.88) [25]. Final scores ranged from 0 to 36, where a higher score indicates dysfunction in problem solving and communication within the family. Scores were standardized and computed as 0 = below the mean and 1 = above the mean.

*Family social support* was assessed at age 14 years, as the questions were omitted from the Cycle 2 data collection. The PMK was asked 6 questions (α = 0.88) derived from the 24 items Social Provisions Scale [26]. Final scores range from 0 to 24, with higher scores indicating the presence of social support to the family. Scores were standardized and computed as 0 = above the mean and 1 = below the mean.

### 2.5. Data Analytic Strategies

Descriptive statistics and analyses were computed using SPSS (v. 28). Long-term prospective associations were then estimated using ordinary least squares regression, stratified by sex. Youth academic achievement indicators at ages 18 and 20 years were linearly regressed on all variables reflecting childhood active leisure. Adjustments for possible omitted variable bias were accounted for by including family characteristics that are either statistically or theoretically linked to the predictor or outcomes.

Analyses were adjusted according to the weight assigned to each participant by Statistics Canada, which was calculated based on their design weight, non-response rate, and post-stratification. Thus, analyses are adjusted to represent the entire population covered by the NLSCY.

#### Missing Data

This study required data from various sources and waves. As expected from a longitudinal study, some participants had incomplete data. “Non-applicable” and “Doesn’t know” answers were coded as missing data. Attrition rate for participants of this study was 27.2% between the predictors and the average grades outcome, and 32.3% between the predictors and the high school completion outcome. Of participants who had data on the predictor, 67.2% did not have data on the average grades outcome and 72.9% did not have data on high school completion. An attrition analysis was conducted to compare participants with incomplete data to participants with complete data at age 12 years on control variables using Pearson Chi-square tests. There were no significant differences on all control variables between participants who had missing data at ages 18 and 20 years and those who had complete data for both boys and girls. We used Multiple Imputation in SPSS v.28 (IBM Statistics, Chicago, IL, USA) to correct for attrition bias in the analyses.

## 3. Results

### 3.1. Descriptive Statistics

Table 1 reports descriptive statistics for all study variables. More boys and girls were taking part in organized sports 1 to 3 times a week (40.1% of boys and 40.2% of girls) and were never taking part in artistic sports (60.0% of boys and 42.8% of girls). More boys were taking part in unstructured physical activity 4 or more times a week (46.2%), whereas more girls were taking part in unstructured physical activity 1 to 3 times a week (39.0%). On a scale from 1 to 6, boys had average grades representing 4.04 and girls, 4.30. Most participants had graduated high school by age 20 years (80.8% of boys and 87.4% of girls).

Table 2 reports adjusted unstandardized regression coefficients reflecting the relationship between concurrent family characteristics and active leisure. For girls, income adequacy and maternal education had the biggest contribution to active leisure, followed by family configuration and family social support. Girls whose family had an inadequate income partook in less unstructured physical activity (β = −0.066, *p* < 0.05) and organized sports (β = −0.087, *p* < 0.01). Girls whose mothers had not graduated from high school engaged in less artistic sports (β = −0.091, *p* < 0.01), and partook in less organized sports when coming from families that were non-intact (β = −0.106, *p* < 0.001), dysfunctional (β = −0.060, *p* < 0.05), or with more social support (β = 0.092, *p* < 0.01). For boys, maternal factors only played a role in engaging in organized sports (maternal depressive symptoms β = 0.071, *p* < 0.05; maternal education β = −0.059, *p* < 0.05).

Table 3 reports the adjusted weighted relationships between childhood active leisure and later academic achievement indicators for boys and girls.

### 3.2. Girls

Compared with girls who participated in less active leisure, girls who participated in more active leisure showed overall better subsequent indicators of academic success. Specifically, more organized sports predicted a 8.2% unit increase on the subsequent average grades scale (*p* < 0.01) and a 7.1% unit increase in likelihood of having received a high school diploma or its equivalent (*p* < 0.05). Artistic sports also predicted a 22.8% unit increase in later average grades (*p* < 0.001). Conversely, unstructured physical activity in girls was associated with a 7.7% decrease in average grades by age 18 years (*p* < 0.01).

### 3.3. Boys

Compared with boys who participated in less active leisure, boys who participated in active leisure had higher chances of subsequently having completed high school. Organized sports predicted a 14.6% unit increase in chances of having received a high school diploma or its equivalent (*p* < 0.001).

## 4. Discussion

While the general importance of practicing physical activity is well known, studies examining the impacts of active leisure have not clearly shown benefits to academic functioning [13]. Results from this study show that having more active leisure time in middle childhood is associated with higher chances of academic achievement in emerging adulthood. It is important that health officials and practitioners encourage families to practice more active leisure, so that children can grow to be successful adults and productive members of the society [14].

Devoting more active leisure time in middle childhood, as compared to less, was generally associated with higher chances of having graduated high school. Practicing sports with a coach was associated with higher chances of having graduated from high school by age 20 years for both boys and girls. Adults who have not completed high school are at risk of substance abuse [27], lower health [28], and higher mortality because of lower incomes [29]. Through academic success, active leisure could then be considered a protective factor to later health.

Practicing sports with a coach or instructor is the type of active leisure that had the most impact on later achievement. This could be explained by the fact that sports are physical activities that require structure, usually by the implementation of rules and objectives [30]. When supervised by an adult and often in teams, sports allow children to develop key skills in various areas—leadership, behaving in a group, prolonged attention—that can be transferred to academic classes [31].

Moreover, for girls, structured active leisure—sports with a coach and artistic sports with an instructor—lead to higher subsequent average grades. These results could be explained by the fact that girls who have greater self-perceptions of competence and self-worth are more likely to take part in sports [32]. These characteristics could also have an influence on academic achievement, which could explain the several associations we found between active leisure in middle childhood and later academic success for girls.

Surprisingly, girls who took part in more physical activity without a coach or instructor had lower grades six years later. These girls chose to spend their leisure time playing games and running, and favored the unstructured environment where rules of play are not as strictly enforced. This could be because popular reasons among adolescent girls not to take part in organized sports are the lack of self-perceived ability and low motivation leading to disengagement, possibly playing a role in school engagement [33]. Thus, according to Human Capital theory, as girls possess fewer athletic skills or perceive themselves as such, it would be more difficult for them to develop new abilities, which could explain why they have lower academic results [13].

A study examining the effects of increasing physical education in school for adolescents found similar results: there were no differences in the academic and cognitive performance of adolescents when increasing the number of physical education sessions from two to four sessions per week, but adolescents who received four high intensity sessions of physical education per week had better cognitive and academic performance than those who received two regular sessions [34]. Thus, it could be that, for girls, unstructured physical activity is not of high enough intensity to influence their academic success.

For boys, only sports with a coach or instructor had an impact on later achievement. Motivation to partake in sports is greatly affected by the instructor for boys [35]. Similarly, teacher enthusiasm has a considerable impact on class engagement for boys [36]. Boys might reap more benefits from practicing physical activity with a positive model, such as a coach, than from doing so with peers or by themselves.

This study is not without limitations. First, this study is observational, and thus cannot imply causal relations. Although our study had incomplete data, attrition bias was reduced by using multiple imputation. To reduce the possibility of confounding effects, we adjusted for baseline academic achievement and multiple family characteristics, increasing internal validity. Data on active leisure and academic achievement was self-reported. Subjective measure tends to be less reliable as they are subject to being overestimated or underestimated. Some of the participants might have had already graduated from high school at age 18 years, in which case their average mark was imputed. This could have impacted the results, as some data was estimated rather than reported by the participants. Future research should focus on using objective reports of active leisure and school averages, for example by using biometric measures and report cards.

The chief strength of this study is its use of a longitudinal design, following participants for nearly a decade and adding temporality to its findings. The stratification of the analyses by sex adds valuable interest to our findings, as boys and girls have important differences in their experience of sports and school. Finally, the data for the first time point of this study was collected in 1996. This historical data offers an excellent opportunity to simulate childhood without the constraints and confounds of ubiquitous technology today. Thus, we can have estimates that are reliable and meaningful. This data still offers challenges in representing with accuracy the modern context, where technology is prominent and might influence how children choose to occupy their free time [37].

The Canadian Paediatric Society recommends outdoor risky play to counter obesity, anxiety, and behavioral difficulties in children [38]. These recommendations align with our findings, suggesting that active play has several long-lasting benefits on child development and adaptation. Parents should be encouraged to lead their children to replace sedentary leisure by playing outside with friends to allow them to fully reap the benefits of an active lifestyle from a young age.

## 5. Conclusions

Above the shift to sedentary leisure, important barriers remain to an active lifestyle in childhood, mainly the financial cost and involvement from parents. Notably, many socio-economic factors, such as family income, family configuration, family dysfunction, and maternal education, had an influence on child active leisure. Structured sports were shown to have long-term benefits on success, reiterating the importance of encouraging play and an active lifestyle throughout childhood. By aiming to reduce the socio-economic barriers that remain to participation in sports, policy officials would help reduce inequities in later access and control over wealth.

## Figures and Tables

**Table 1:** Descriptive statistics for study variables.

	Boys			Girls		
	*M (SD)*	*Categorical Variables (%)*	*Range*	*M (SD)*	*Categorical Variables (%)*	*Range*
*Predictors (age 12 years)*						
Organized sports	2.77 (1.06)		1–4	2.62 (1.08)		1–4
1 = never		18.8			23.9	
2 = less than once a week		13.2			13.1	
3 = 1 to 3 times a week		40.1			40.2	
4 = 4 or more times a week		28.0			22.9	
Artistic sports	1.74 (1.01)		1–4	2.11 (1.08)		1–4
1 = never		60.0			42.8	
2 = less than once a week		13.5			13.3	
3 = 1 to 3 times a week		19.1			33.6	
4 = 4 or more times a week		7.4			10.3	
Unstructured physical activity	3.12 (1.01)		1–4	2.81 (1.01)		1–4
1 = never		11.0			14.9	
2 = less than once a week		12.3			17.4	
3 = 1 to 3 times a week		30.5			39.0	
4 = 4 or more times a week		46.2			28.7	
*Outcomes*						
Average grades (age 18 years)	4.04 (0.59)		1–6	4.30 (0.65)		1–6
High school completion (age 20 years)						
0 = yes		80.8			87.4	
1 = no		19.2			12.6	
*Control variables (age 12 years)*						
Family configuration						
1 = non-intact		29.0			30.0	
Income adequacy						
1 = inadequate		12.6			12.3	
Maternal education						
1 = did not graduate high school		17.0			16.7	
Maternal depressive symptoms						
1 = depression symptoms above average		35.3			31.7	
Family dysfunction						
1 = dysfunctional family		55.6			55.0	
Family social support						
1 = social support below average		43.4			42.8	
Academic achievement						
1 = performance below average		14.2			12.5	

Notes. Analyses corrected for attrition bias. Data were compiled from the final master file of the National Longitudinal Survey of Children and Youth, Cycles 2–8 (1994–2009), ©Statistics Canada.

**Table 2:** Unstandardized weighted regression coefficients (standard error) reflecting the adjusted relationship between family characteristics at age 12 years and extracurricular exercise at age 12 years, for boys and girls.

	Organized Sports		Artistic Sports		Unstructured Physical Activity	
	*Boys*	*Girls*	*Boys*	*Girls*	*Boys*	*Girls*
Family configuration	−0.13 (0.07)	−0.24 (0.07) ***	0.10 (0.07)	−0.08 (0.07)	0.01 (0.07)	−0.08 (0.06)
Income adequacy	−0.13 (0.10)	−0.29 (0.10) **	−0.01 (0.09)	−0.14 (0.10)	0.08 (0.09)	−0.20 (0.09) *
Maternal education	−0.17 (0.08) *	−0.15 (0.09)	−0.10 (0.08)	−0.28 (0.09) **	0.01 (0.08)	0.07 (0.08)
Maternal depressive symptoms	0.15 (0.06) *	−0.06 (0.07)	0.08 (0.06)	−0.06 (0.07)	0.10 (0.06)	−0.08 (0.06)
Family dysfunction	−0.05 (0.06)	−0.13 (0.06) *	−0.03 (0.06)	−0.02 (0.06)	−0.10 (0.06)	0.03 (0.06)
Family social support	0.03 (0.06)	0.20 (0.06) **	−0.05 (0.06)	0.001 (0.06)	0.01 (0.06)	0.02 (0.06)
R^2^	0.01 *	0.05 ***	0.01	0.02 **	0.01	0.01 *

Notes. * *p* < 0.05, ** *p* < 0.01, *** *p* < 0.001. Analyses corrected for attrition bias. Data were compiled from the final master file of the National Longitudinal Survey of Children and Youth, Cycles 2–8 (1994–2009), ©Statistics Canada.

**Table 3:** Unstandardized weighted regression coefficients (standard error) reflecting the adjusted relationship between extracurricular sports at age 12 years and school achievement at ages 18 and 20 years, for boys and girls.

		Age 18 Years	Age 20 Years
		*Average Grades*	*High School Completion*
**Boys, age 12 years**	Organized sports	−0.002 (0.02)	−0.06 (0.01) ***
	Artistic sports (dance, gymnastics, etc.)	−0.01 (0.02)	0.01 (0.01)
	Unstructured physical activity	0.002 (0.02)	−0.02 (0.01)
	Family configuration	0.13 (0.04) **	0.21 (0.03) ***
	Income adequacy	−0.04 (0.06)	0.28 (0.04) ***
	Maternal education	0.20 (0.05) ***	0.17 (0.03) ***
	Maternal depressive symptoms	0.10 (0.04) *	−0.05 (0.02) *
	Family dysfunction	0.03 (0.04)	−0.03 (0.02)
	Family social support	−0.17 (0.04) ***	0.10 (0.02) ***
	Academic achievement	−0.000 (0.05)	0.08 (0.03) **
	R^2^	0.05	0.25 ***
**Girls, age 12 years**	**Organized sports**	−0.05 (0.02) **	−0.02 (0.01) *
	**Artistic sports (dance, gymnastics, etc.)**	−0.14 (0.02) ***	**−0.01 (0.01)**
	**Unstructured physical activity**	0.05 (0.02) **	**−0.004 (0.01)**
	Family configuration	0.08 (0.04) *	0.13 (0.02) ***
	Income adequacy	0.07 (0.06)	0.15 (0.03) ***
	Maternal education	0.18 (0.06) **	0.19 (0.03) ***
	Maternal depressive symptoms	0.18 (0.04) ***	0.06 (0.02) **
	Family dysfunction	0.01 (0.04)	0.02 (0.02)
	Family social support	−0.09 (0.04) *	0.07 (0.02) ***
	Academic achievement	0.18 (0.05) **	0.07 (0.03) *
	R^2^	0.15 ***	0.19 *

Notes. * *p* < 0.05, ** *p* < 0.01, *** *p* < 0.001. Analyses corrected for attrition bias. A higher score on outcome variables indicates a child that is at risk (lower grades, no high school diploma). Data were compiled from the final master file of the National Longitudinal Survey of Children and Youth, Cycles 2–8 (1994–2009), ©Statistics Canada.

## Data Availability

The data presented in this study are available on request from Statistics Canada. The data are not publicly available due to permission of Statistics Canada.
